# Correlation of non-auditory comorbidities and hearing loss in chronic subjective tinnitus patients: a retrospective database study

**DOI:** 10.3389/fneur.2025.1596274

**Published:** 2025-06-26

**Authors:** Konstantin Tziridis, Benjamin Neubert, Anna Seehaus, Patrick Krauss, Achim Schilling, Petra Brüggemann, Birgit Mazurek, Holger Schulze

**Affiliations:** ^1^Experimental Otolaryngology, University Hospital Erlangen, Waldstrasse, Germany; ^2^Computational Cognitive Neuroscience (CCN) Group, Pattern Recognition Lab, Friedrich-Alexander University Erlangen-Nürnberg, Erlangen, Germany; ^3^Tinnitus Center, Charité – Universitätsmedizin Berlin, Corporate Member of Freie Universität Berlin and Humboldt-Universität zu Berlin, Berlin, Germany

**Keywords:** chronic subjective tinnitus, comorbidities, audiometry, retrospective patient study, hearing loss

## Abstract

**Background:**

Tinnitus is a symptom often associated with hearing loss (HL). Its development and progression are still not completely clear, as the heterogeneity of tinnitus-related HL data is high. Here, we attempt to investigate whether a part of this variance can be correlated with single or combinations of non-auditory comorbidities using pure-tone audiometric data in a collective of chronic subjective tinnitus patients.

**Methods:**

The information of 136 tinnitus patient files was extracted retrospectively. The patients did not suffer from any auditory impairment except a possible HL and tinnitus; non-auditory comorbidities were identified from the files and categorized by their ICD-10 category. Comorbidity classes were endocrine/metabolic diseases, psychiatric/behavioral disorders, diseases of the central nervous system, diseases of the circulatory system, diseases of the respiratory system, diseases of the digestive system, and muscle-skeletal diseases. The pure-tone audiometry data, as well as tinnitus pitch and loudness, were correlated with their non-auditory comorbidity classes and patients’ age group using non-parametric and parametric analyses, where appropriate.

**Results:**

Depending on the age group, the number of comorbidities could lead to a significant increase or decrease in HL. Only in older patients, a linear correlation between the number of non-auditory comorbidities and an increase in HL could be found. Moreover, the correlation between maximal HL frequency and tinnitus frequency can only be seen in specific age and comorbidity-number groups. Only some specific non-auditory comorbidity classes showed significant effects (decrease or increase) on HL in specific age groups.

**Conclusion:**

Taken together, we argue that in future tinnitus patient studies, non-auditory comorbidities should be taken into account as possible covariables that might explain the variance found in the auditory threshold development of these patients.

## Introduction

Tinnitus—the percept of sound without a physical source—is a symptom often associated with hearing loss (HL) but can also occur without it (1–4). In many cases, the cause of the HL is of central origin (sensorineural hearing loss, SNHL) but can also be conductive (5). Tinnitus is often a more burdening problem than the HL itself (6, 7).

It is still unclear how tinnitus develops and chronifies exactly, even though several models try to explain these mechanisms from different angles (8–13). These SNHL-based models mainly try to explain the underlying neurophysiological mechanisms of tinnitus development and/or chronicity with different assumptions and resulting predictions. From the comparison of the model’s predictions and the clinical reality, one can deduce the validity of the model for most tinnitus patients, namely those with a tonal tinnitus percept. When prediction and reality fit together well, the model might explain the underlying neurophysiological mechanisms and provides insights toward new therapies.

Unfortunately, due to the individual and very complex pathologies of tinnitus and, still, a lack of knowledge, there is no therapy targeting the cause of tinnitus yet. Nevertheless, several such model-based treatment approaches have been developed and applied with more or less success recently (14–19). Restoration of hearing (20–22) or modulation of the neuronal processing of auditory signals [e.g. (17, 19)] are such approaches that lead to a reduction of the tinnitus loudness and in consequence to a reduction of the tinnitus associated burden.

One of the major problems of all studies with tinnitus patients—and therefore the comparison with model predictions and the underlying mechanisms—is the huge variability in the data (23–25).

This heterogeneity of tinnitus is also reflected in the patients’ pure-tone audiometry, an easily accessible measurement that allows inference to the pathological state of the auditory system. The heterogeneity is, on the one hand, a trivial problem, as tinnitus can be a symptom of different forms of HL—either SNHL, conductive, or even inherited (26)—and is therefore also dependent on cofactors such as the patients’ age (e.g., the occurrence of presbycusis). On the other hand, other diseases (e.g., Meniere’s disease) of the auditory system or non-auditory comorbidities can influence HL and/or the occurrence of tinnitus (27–31) and therefore add further variance to the patients’ data. All these different covariables make the measurement of “baseline” tinnitus data, the comparison with the models, and successively the changes during treatments difficult and hard to interpret.

Several studies already assessed the problem of tinnitus heterogeneity from various points of view [e.g. (23, 25)]. Many of these investigations focus on one or a few comorbidities and the specific effects that these disease can have on hearing and tinnitus perception. In many cases, the underlying neuronal and/or metabolic mechanisms are still unclear, even though some of these specific diseases affect major parts of the population.

This is, for example, the case with diabetes mellitus, where the higher occurrence of sensorineural hearing loss with diabetes types 1 and 2 is well-documented, but the exact neuropathic mechanism is still under debate (32). Furthermore, in cardiovascular diseases, the risk of developing hearing loss and associated conditions such as tinnitus is enhanced, but the exact relationship has only been addressed by a few studies (33).

One possible way to explain tinnitus and develop new treatment strategies (19, 34) is the investigation of the neurophysiological basis of tinnitus development and chronification in the animal model (35, 36). As soon as the translational step to human patients is taken, one is confronted with the problem of the heterogeneity of tinnitus. Then, it becomes clear that the heterogeneity of this symptom of HL has to be taken into account for a successful treatment strategy. In this retrospective study, the problem of tinnitus heterogeneity is approached with regard to general health rather than specific neurophysiologic mechanisms. Possible correlations between different single or combinations of non-auditory comorbidities and pure-tone air conductance hearing levels were investigated in patients who primarily came to the ENT hospital in Erlangen because of their suffering from tinnitus and without any severe auditory comorbidities except for a possible HL (independent of the type of HL). Our hypothesis is that a part of the variance of audiometric data of tinnitus patients of different age groups can be explained by the presence of different non-auditory comorbidities.

## Methods

### Study design and ethics statement

A retrospective study on anonymized audiometric data (pure-tone air conductance hearing loss, tinnitus frequency, and tinnitus loudness) from medical files of tinnitus patients of the ENT hospital in Erlangen from 2000 and 2018 was performed. By signing the treatment contract, all patients gave their consent that their data could be used for scientific purposes. Therefore, no further declaration of consent or ethics committee vote was necessary. All research was conducted in accordance with the Declaration of Helsinki, and the reporting followed the STROBE guidelines.

### Inclusion and exclusion criteria

Inclusion criteria for the files of chronic subjective tinnitus patients were that the patients’ complained about tinnitus as the main reason for coming to the clinic and an age of at least 18 years at the time of medical examination. The subjective tinnitus percept had to be pure-tone or narrowband to determine a tinnitus frequency. Exclusion criteria were objective tinnitus, acute auditory diseases (with the exception of hearing loss, independent of its type), or recent surgeries affecting the auditory system, as well as hyperacusis, as assessed by the German version of the hyperacusis questionnaire (37). This resulted in a patient collective of 136 adult tinnitus patients.

### Non-auditory comorbidity categories

All information on the auditory and non-auditory comorbidities was extracted from the patient’s files. In most cases, the information was based on data from physicians’ diagnoses included in the files. Only a minority of the information (ca. 15%) was based on patient’s own statements. Acute (e.g., asthma bronchiale/allergies, *N* = 6) and chronic (e.g., diabetes mellitus, type 1 or 2 *N* = 8 or hypothyreosis *N* = 23) non-auditory comorbidities of the patients—independent of individual treatment status—were taken into account and separated into seven categories according to the ICD-10-GM codes. Category 1: Endocrine system/metabolic diseases. This included, e.g., diabetes mellitus or hypothyroidism. Category 2: Psychiatric/behavioral disorders. This included, e.g., clinical depression or anxiety. Category 3: diseases of the central nervous system. This included, e.g., strokes or space-occupying tumors of the brain. Category 4: Diseases of the circulatory system. This included, e.g., patients who suffered from hypertension, earlier cardiac arrests, or arteriosclerosis. Category 5: Diseases of the respiratory system. This included, e.g., chronic obstructive pulmonary disease or chronic asthma bronchiale. Category 6: Diseases of the digestive system. This included, e.g., inflammatory bowel disease or ulcerative colitis. Category 7: Muscle-skeletal diseases. This included, e.g., disc-related spinal disorders, arthritis, or osteoporosis.

### Outcome measures

The mean pure-tone air conduction hearing levels of the patient’s tinnitus-affected ears (data of all ears were available but selected for the phantom percept) as well as tinnitus frequency (often also referred to as tinnitus pitch, which is an interchangeable wording) and loudness (calculated relative to the hearing level in dB SL) were investigated depending on the non-auditory comorbidity category and patients’ age category (cf. below). Note that bone conductance data was not included, as it was not differentiated between SNHL or conductive HL in this study. The pure-tone audiometry, as well as the tinnitus frequency and loudness determination, were performed using standardized audiometric testing instruments of the audiological clinic. In brief, the hearing levels were obtained via an audiometer with automatic hearing level detection algorithms from the patients wearing earphones in a soundproof chamber. All devices fulfilled the necessary requirements according to ISO 8253-1 and 8,253-3. Air conduction hearing level thresholds were measured for both ears separately for every patient. Analyzed frequencies were 250, 500, 750, 1,000, 1,500, 2000, 3,000, 4,000, 6,000, and 8,000 Hz, and HL was calculated as the difference from the normal pure-tone audiometry (range: −10 dB to 130 dB). Tinnitus frequency and loudness were measured using the same devices by asking the patients to match their percept to the presented frequencies and stimulus intensities.

### Data evaluation and statistics

For the statistical data analysis, Statistica 14 (TIBCO Software, Palo Alto, CA, USA) was used. The patients were categorized as in earlier studies according to their age at the time of performed measurements, as extracted from their clinical files and calculated in the decimal system with one digit precision into young: 18 to 39.9 years, middle-aged: 40 to 59.9 years, and older adults: 60 + years. The tinnitus-related data (tinnitus frequency and loudness), as well as the number of comorbidities, were analyzed by non-parametric statistics. Unreported comorbidities from the patient files could not be included in the statistics and were therefore not counted. For group comparisons, Mann–Whitney U-tests or Kruskal–Wallis ANOVAs were used. The 45 (33%) tinnitus patients without any non-auditory comorbidities were seen as “control tinnitus patients without comorbidities,” to which the other comorbidity groups could be compared. In the young patients group, only data from patients with one non-auditory comorbidity could be compared to them. In the two other age groups, we could compare the data of patients with one to five non-auditory comorbidities to those of the “control tinnitus patients.” The pure-tone audiometry data [i.e., the HL (dB)] of the patient’s tinnitus-affected ears were analyzed using parametric statistics, as the normal distribution assumption was not rejected (Shapiro–Wilk test, W = 0.96, *p* = 0.10). All HL-related data of the tinnitus-affected ears of the patients were analyzed using two-factorial ANOVAs with Tukey post-hoc tests, corrected for multiple testing using the mean and 95% confidence interval for testing and visualization. For a better interpretation of the results, the effect size of the ANOVA results is presented with the Partial Eta-Squared values (η^2^) with values of η^2^ = 0.01, η^2^ = 0.06, and η^2^ = 0.16, representing small, medium, and large effect thresholds, respectively. The distance (D) of the tested audiometric frequency (AF in Hz) to the tinnitus frequency (TF in Hz) was calculated by Equation 1.


(1) D=log2(AF/TF).

It is rounded to the nearest integer. This was used to align the measured HL to the determined TF. Note that for the analyses, only D values ranging from −4 to +2 octaves relative to the TF were used, as D values beyond this range were rare in the patient collective. This approach was already used in earlier studies to focus the analyses on the part with the highest data saliency (34).

## Results

### Patient collective and comorbidities

The 136 adult tinnitus patients (56 ♀, 80 ♂) included in this study had a mean age ± standard deviation of 50.9 ± 15.5 years. Female (51.3 ± 15.5 years) and male patients (50.6 ± 15.5 years) did not differ significantly in their mean age (Student’s *t*-test, *p* = 0.79). The adult patient collective was divided into three age categories: young (<40 years; *n* = 18; mean age (± standard deviation): 23.9 ± 3.6 years), middle-aged (40–59.9 years; *n* = 85; 48.8 ± 7.6 years), and older adults (60 + years; *n* = 33; 70.6 ± 7.6 years) patients to account for age-related hearing changes and comorbidity probability. Earlier middle ear surgeries were reported in 5 out of 136 (3.7%) patients (e.g., tympanoplasty surgeries). Hearing aids were used by 5 out of 136 (3.7%) patients (one monaural and four binaural). A mean hearing loss of 20 dB or more was diagnosed in 36 out of 136 (26.5%) patients; in 7 out of 136 (5.1%) cases, a common cold was mentioned that could change the hearing ability to a certain degree; nevertheless, none of these patients showed any active middle ear disorders (normal acoustic reflexes). Finally, 46 out of 136 (33.8%) patients mentioned earlier episodes of vertigo. The patients reported either unilateral (young: *n* = 13; middle-aged: *n* = 38; older adults: *n* = 19) or bilateral (young: *n* = 5; middle-aged: *n* = 47; older adults: *n* = 14) pure-tone (young: *n* = 12; middle-aged: *n* = 56; older adults: *n* = 21) or narrowband noise tinnitus (young: *n* = 6; middle-aged: *n* = 28; older adults: *n* = 11). Additionally, two patients (one middle-aged and one older adult) reported pure-tone in one ear and narrowband noise in the other ear. All other patients reported only one kind of tinnitus percept with a determinable center frequency, even though in the case of a binaural percept, the frequency could differ. Only the pure-tone audiometries of the patient’s tinnitus-affected ears were used for the analyses described below, so different center frequencies of both ears did not affect the evaluation.

The patient’s TF in the three age groups was not significantly different from each other [median frequency (interquartile range): young: 4000 Hz (750 Hz, 8,000 Hz); middle-aged: 4000 Hz (2000 Hz, 6,000 Hz); older adults: 4000 Hz (1,000 Hz, 6,000 Hz); Kruskal–Wallis ANOVA: H(2, 202) = 2.30, *p* = 0.32]. The same was true for the tinnitus loudness, given in dB sensation level (dB SL); here, the values of the median loudness with the interquartile range are also provided [young: 7 dB SL (0 dB SL, 10 dB SL); middle-aged: 7.5 dB SL (−3 dB SL, 15 dB SL); older adults: 10 dB HL (2 dB SL, 19 dB SL); Kruskal–Wallis ANOVA: H (2, 196) = 2.97, *p* = 0.23]. Nevertheless, patients in the three age categories showed a significantly different number [Χ^2^ (20, 136) = 13.58, *p* = 0.035] of non-auditory comorbidities (the exact patient numbers for each category are given in Table 1); these are summarized in Table 2 (patients could suffer from more than one non-auditory comorbidity: 169 comorbidities in 136 tinnitus patients).

**Table 1:** Overview of non-auditory comorbidity categories in patients separated by age group.

Comorbidity category	Young patients	Middle-aged patients	Older adults patients	Σ of all patients
Endocrine system/metabolic diseases	1/18 (5.5%)	24/85 (28.2%)	15/33 (45.5%)	**40/136 (29.4%)**
Psychiatric/behavioral disorders	4/18 (22.2%)	20/85 (23.5%)	4/33 (12.1%)	**28/136 (20.6%)**
Diseases of the central nervous system	0/18	6/85 (7.1%)	3/33 (9.1%)	**9/136 (6.6%)**
Diseases of the circulatory system	0/18	22/85 (25.9%)	19/33 (57.6%)	**41/136 (30.1%)**
Diseases of the respiratory system	0/18	5/85 (5.9%)	5/33 (15.1%)	**10/136 (7.4%)**
Diseases of the digestive system	0/18	9/85 (10.6%)	1/33 (3.0%)	**10/136 (7.4%)**
Muscle-skeletal diseases	1/18 (5.5%)	19/85 (22.4%)	11/33 (33.3%)	**31/136 (22.8%)**
**Σ All comorbidity classes**	**6**	**105**	**58**	**169 comorbidities in 136 patients**

Bold values represent the sum over all patients in the specific groups.

**Table 2:** Number of non-auditory comorbidities in 136 tinnitus patients separated by age group.

Age group	0 Comorbidities	1 Comorbidity	2 Comorbidities	3 + Comorbidities
Young	11 (8.1%)	7 (5.1%)	0	0
Middle	28 (20.6%)	25 (18.4%)	20 (14.7%)	12 (8.8%)
Older adults	6 (4.4%)	11 (8.1%)	7 (5.1%)	9 (6.6%)

Trivially, with increasing age, the number of non-auditory comorbidities rose significantly [Kruskal–Wallis ANOVA, H (3, 136) = 14.93, *p* < 0.001], and multiple comparisons of means post-hoc tests showed that young patients showed significantly fewer non-auditory comorbidities than middle-aged (*p* = 0.015) or older adults patients (*p* < 0.001), while those two last groups did not show significant differences in comorbidity numbers (*p* = 0.25). When analyzing the tinnitus frequency with these non-auditory comorbidity categories in the three age groups independently, we also did not find any significant differences between the median frequency of patients with different numbers of comorbidities (young: 0 comorbidities: 1500 Hz (500 Hz, 8,000 Hz), 1 comorbidity: 6000 Hz (4,000 Hz, 8,000 Hz), Mann–Whitney U-test, *p* = 0.21; middle-aged: 0 comorbidities: 4000 Hz (2000 Hz, 6,000 Hz), 1 comorbidity: 6000 Hz (4,000 Hz, 6,000 Hz), 2 comorbidities: 4000 Hz (2000 Hz, 6,000 Hz), 3 + comorbidities: 4000 Hz (2000 Hz, 6,000 Hz), Kruskal–Wallis ANOVA, H (3, 132) = 6.12, *p* = 0.11; older adults: 0 comorbidities: 3000 Hz (1,000 Hz, 4,000 Hz), 1 comorbidity: 4000 Hz (500 Hz, 8,000 Hz), 2 comorbidities: 4000 Hz (2000 Hz, 6,000 Hz), 3 + comorbidities: 3000 Hz (1,500 Hz, 6,000 Hz), Kruskal–Wallis ANOVA, H (3, 47) = 0.94, *p* = 0.82). This is also true for the tinnitus loudness (young: 0 comorbidities: 8.5 dB SL (1 dB SL, 13 dB SL), 1 comorbidity: 6 dB SL (1 dB SL, 7 dB SL), Mann–Whitney U-test, *p* = 0.48; middle-aged: 0 comorbidities: 3.5 dB SL (2 dB SL, 11.5 dB SL), 1 comorbidity: 7 dB SL (−4 dB SL, 13 dB SL), 2 comorbidities: 7.5 dB SL (3.5 dB SL, 16 dB SL), 3 + comorbidities: 3.5 dB SL (−1 dB SL, 10 dB SL), Kruskal–Wallis ANOVA, H (3, 128) = 3.70, *p* = 0.30; older adults: 0 comorbidities: 14 dB SL (8 dB SL, 19.5 dB SL), 1 comorbidity: 3 dB SL (−3 dB SL, 13 dB SL), 2 comorbidities: 8 dB SL (3 dB SL, 25 dB SL), 3 + comorbidities: 13 dB SL (5 dB SL, 19.5 dB SL), Kruskal–Wallis ANOVA, H (3, 46) = 3.54, *p* = 0.32). In other words, neither tinnitus frequency nor tinnitus loudness did show any dependency on the patient’s age.

### Correlation of the audiometric data of tinnitus patients with and without the presence of non-auditory comorbidities

**Figure 1:**
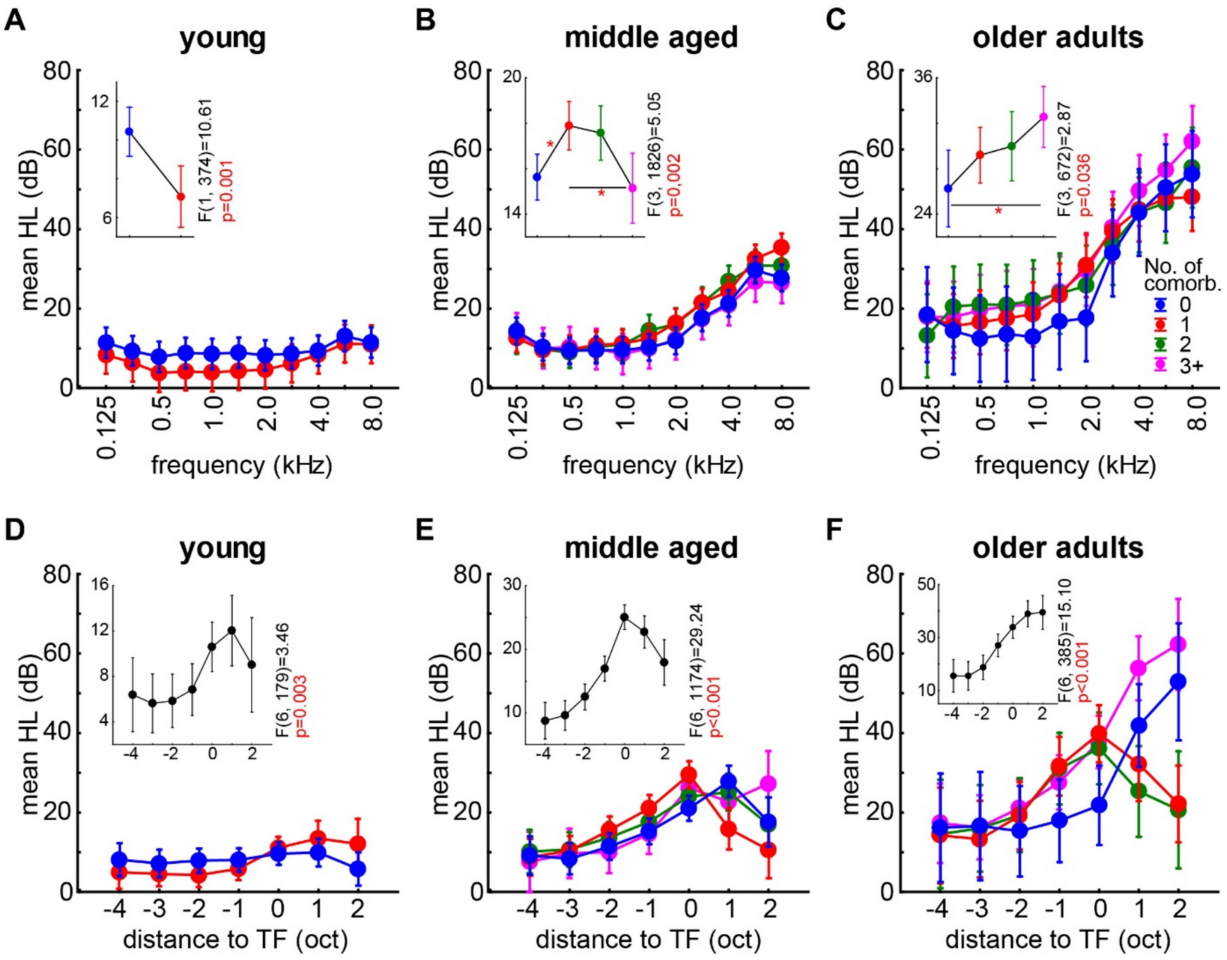
Mean hearing level dependent on the number of non-auditory comorbidities for the three tinnitus patients age groups. **(A)** Interaction plot of two-factorial ANOVA of the mean HL (dB) in young tinnitus patients (*n* = 18) dependent on the factors frequency and number of non-auditory comorbidities (color-coded, ref. to **C**). The inset depicts the one-factorial part of HL analysis [mean HL (dB)] with the factor number of non-auditory comorbidities; statistics are given on the side. **(B,C)** Same plots as described above for middle-aged (*n* = 85) and older adult tinnitus patients (*n* = 33), respectively. Asterisks in insets depict the level of significant Tukey post-hoc tests, *p < 0.05. **(D–F)** Interaction plots of two-factorial ANOVAs on mean HL (dB) in tinnitus patients of different age groups dependent on the factors distance to tinnitus frequency (TF) given in octaves and number of non-auditory comorbidities. Color codes as above. The inset depicts the one-factorial part of HL analysis [mean HL (dB)] with the factor distance to TF; statistics are given on the side. For details on the analyses, see the text.

In the first analyses, the pure-tone air-conductance audiometric HL of the patients of the three age groups was assessed independently by two-factorial ANOVAs with the factors *stimulation frequency* and *number of non-auditory comorbidities*. As mentioned above, for the young patients group, in the factor *number of non-auditory comorbidities*, only the comparison between zero and one comorbidity was possible. The results are summarized in Figure 1; in the text, significant values are marked by asterisks. In all patient groups, a significant effect of *stimulation frequency* on the HL was found [not shown in Figure; young: *F* (10, 374) = 1.92, *p* = 0.041 *, η^2^ = 0.049 (*medium effect*); middle-aged: *F* (10, 1826) = 59.49, *p* < 0.001 *, η^2^ = 0.246 (*large effect*); older adults: *F* (10, 672) = 35.90, *p* < 0.001 *, η^2^ = 0.348 (*large effect*)]. In other words, higher frequencies were more affected than lower frequencies. We also found a dependency of the HL on the *number of non-auditory comorbidities*. In the young tinnitus patients, the mean HL was significantly weaker with one of these comorbidities [Figure 1A**, inset**; *F* (1, 374) = 10.61, *p* = 0.001 *, η^2^ = 0.028 (*medium effect*)], while the middle-aged tinnitus patients did show a significantly increased HL with one non-auditory comorbidity [Figure 1B**, inset**; *F* (3, 1826) = 5.05, *p* = 0.002 *, η^2^ = 0.008 (*no effect*)]. Older adult tinnitus patients showed, on average, a significantly more severe HL only with at least three non-auditory comorbidities [Figure 1C**, inset**; *F* (3, 672) = 2.87, *p* = 0.036 *, η^2^ = 0.013 (*small effect*)], but at a generally higher level than the other two age group patients with the above-mentioned more severe HL at higher frequencies. No interaction of the two factors (*stimulation frequency* and *number of comorbidities*) could be found in all three age groups [Figures 1A–C; young: *F* (10, 374) = 0.22, *p* = 0.99, η^2^ = 0.006 (*no effect*); middle-aged: *F* (10, 1826) = 0.69, *p* = 0.90, η^2^ = 0.011 (*small effect*); older adults: F (10, 672) = 0.39, *p* = 0.99, η^2^ = 0.017 (*small effect*)]. In other words, dependent on the tinnitus patients’ age and number of non-auditory comorbidities, the hearing thresholds can be significantly different, even if no further auditory comorbidities are present. Note that only in the older adult tinnitus patients, we found a steadily increasing mean HL with an increasing number of comorbidities, while in the middle-aged patients, this dependency was non-linear and in young patients even inverted (at least in the two data points available).

It is well-known that the maximum of the HL and the TF correlate with each other [e.g. (38)]. Usually, the TF is found to be at the frequency of the maximum HL, but comorbidities and/or age might have an influence on this correlation. Therefore, we analyzed the mean HL dependent on the *distance of the tested frequency to the determined tinnitus frequency* (D, cf. equation 1; given in octaves) and the *number of non-auditory comorbidities* as factors in independent ANOVAs for each age group. For better comparison, we used only the distance range of −4 to +2 oct relative to the TF, which included 81.5% (1812/2222 data points) of the complete HL data provided above. The reasoning behind these analyses was—as mentioned above—to investigate if the HL is dependent on the *distance to the TF* and if this dependency is further dependent on the *number of non-auditory comorbidities*. We found, first, that in young tinnitus patients, the pathophysiological models of tinnitus predicted a significant dependency of the HL on the *distance to TF* [Figure 1D**, inset**; *F* (6, 179) = 3.46, *p* = 0.003 *, η^2^ = 0.10 (*medium effect*)] with the maximum HL at +1 oct relative to the TF (Tukey post-hoc tests, *p* < 0.05). In the factor *number,* no difference between HL without any non-auditory comorbidities and one comorbidity was found [not shown in Figure; *F* (1, 179) = 0.002, *p* = 0.97, η^2^ = 0.00001 (*no effect*)] and no significant interaction was found [Figure 1D, *F* (6, 179) = 1.74, *p* = 0.11, η^2^ = 0.055 (*small effect*)]. Second, in the middle-aged tinnitus patients, the predicted dependency of HL and *distance to TF* was again found [Figure 1E**, inset**; *F* (6, 1,174) = 29.24, *p* < 0.001, η^2^ = 0.13 (*medium effect*)] with the maximum HL at the TF (Tukey post-hoc tests, *p* < 0.001). No difference in mean HL between the *number of non-auditory comorbidities* was found [not shown in Figure; *F* (3, 1,174) = 0.38, *p* = 0.77, η^2^ = 0.0009 (*no effect*)], but we found a significant interaction of both factors [Figure 1E; *F* (18, 1,174) = 2.42, *p* < 0.001 *, η^2^ = 0.036 (*small effect*)]. Tukey *post-hoc* tests indicated that especially the HL of the tinnitus patients without any non-auditory comorbidities showed a higher peak TF location compared to the other comorbidity groups (range between *p* = 0.001 and *p* = 0.047). Finally, in the older adults age group, we found a sigmoidal-shaped dependency of the HL on the *distance to the TF* [Figure 1F**, inset**; *F* (6, 385) = 15.10, *p* < 0.001 *, η^2^ = 0.19 (*large effect*)] with a maximum at the upper end of the investigated range of +2 oct relative to TF (Tukey *post-hoc* tests, *p* < 0.001). We also found a significant dependency on the *number of non-auditory comorbidities* [not shown in Figure; *F* (3, 385) = 7.63, *p* < 0.001 *, η^2^ = 0.056 (*medium effect*)] with the “3+” category having significantly higher HL values compared to two of the other three categories (Tukey *post-hoc* tests, “0 vs. 3+” and “2 vs. 3+,” *p* < 0.05 *; “1 vs. 3+,” *p* = 0.075). The interaction of both factors (*distance to TF* and the *number of comorbidities*) did show a significant value as well [Figure 1F; *F* (18, 385) = 3.48, *p* < 0.001 *, η^2^ = 0.14 (*medium effect*)]. Here, the peak of the HL was at the TF for one and two non-auditory comorbidities, while for the two extreme cases (“0” and “3+”), the maximum HL was found at +2 oct relative to the TF (Tukey post-hoc tests, *p* < 0.001 *). In other words, the statement that the maximum HL can be found at or around the TF is only partially true and depends on the factor of age and, more importantly, also on the number of non-auditory comorbidities. Again, no “simple” linear correlation of the data with the number of non-auditory comorbidities could be identified.

With this knowledge, we aimed to identify the possible non-auditory comorbidity (or comorbidities) that affected the HL in the different tinnitus patient age groups most. The non-auditory comorbidities were separated into seven categories (cf. Methods).

The patient’s mean HL of the different age groups was then analyzed by independent two-factorial ANOVAs with the factors *stimulation frequency* and n*on-auditory comorbidity presence* (i.e., with or without the specific comorbidity). The results of all analyses are given in Table 3. Note that analyses were not possible in all age groups dependent on the specific non-auditory comorbidity; we refrained from using the data of the young patients completely (cf. Table 1). From the analyses, it became obvious that frequency-dependent HL was mostly independent of the non-auditory comorbidity in middle-aged and older adult tinnitus patients, as in all cases, the “standard” HL-pattern of low HL in lower frequencies and higher HL in higher frequencies was either significant or showed a tendency (column frequency in Table 3). The analyses of the factor comorbidity presence resulted in a more differentiated picture. In the case of diseases of the endocrine system/metabolism, patients of these two age groups with enough data for analysis showed significantly higher HL without that non-auditory comorbidity compared to patients with that specific comorbidity. In the remaining six categories of non-auditory comorbidities, middle-aged tinnitus patients showed higher HL with the specific comorbidity in two categories (digestive and muscle-skeletal system); older adult patients showed this only with circulatory system comorbidities. Finally, we found interactions of both factors (i.e., frequency and presence of a comorbidity) only in one case, namely in middle-aged tinnitus patients with or without muscle-skeletal system comorbidities. In other words, in all other cases with significant differences in mean HL dependent on the non-auditory comorbidity, the whole pure-tone audiometry was shifted in a parallel manner, which was not the case in this specific group.

**Table 3:** F-statistics results of two-factorial ANOVAs of mean HL ± 95% confidence interval (dB).

Comorbidity category	Age group	Frequency	Comorbidity presence	Interaction
Endocrine system/metabolism	Middle	*F*(10, 1,144) = 7.64, *p* < 0.001; η^2^ = 0.063	*F*(1, 1,144) = 7.69 *p* = 0.006, η^2^ = 0.007 **with** (24): 12.97 (±2.77) dB **without** (61): 17.00 (±0.78) dB	*F* (10, 1,144) = 0.73, *p* = 0.70; η^2^ = 0.006
	Older	*F*(10, 694) = 9.64, *p* < 0.001; η^2^ = 0.30	*F*(1, 694) = 4.55 *p* = 0.034; η^2^ = 0.04 **with** (15): 20.73 (±5.72) dB **without** (18): 27.51 (±2.67) dB	*F* (10, 694) = 0.14, *p* = 0.99; η^2^ = 0.009
Psychiatric/behavioral	Middle	*F*(10,1,144) = 16.06, *p* < 0.001; η^2^ = 0.123	*F*(1, 1,144) = 1.11 *p* = 0.29, η^2^ = 0.001 **with** (20): 15.44 (±2.47) dB **without** (65): 16.74 (±0.69) dB	*F* (10, 1,144) = 0.40, *p* = 0.95; η^2^ = 0.004
	Older	*F*(10, 694) = 3.75, *p* < 0.001; η^2^ = 0.14	*F*(1, 694) = 0.02 *p* = 0.88, η^2^ = 0.0003 **with** (4): 27.05 (±9.91) dB **without** (29): 26.26 (±2.49) dB	*F* (10, 694) = 0.07, *p* = 0.99; η^2^ = 0.005
Central nervous system	Middle	*F*(10, 1,144) = 9.19, *p* < 0.001; η^2^ = 0.074	*F*(1, 1,144) = 0.87 *p* = 0.35, η^2^ = 0.0008 **with** (6): 18.17 (±3.16) dB **without** (79): 16.61(±0.77) dB	*F* (10, 1,144) = 0.06, *p* = 0.99; η^2^ = 0.0005
	Older	*F*(10, 694) = 12.53, *p* < 0.001; η^2^ = 0.153	*F*(1, 694) = 1.96 *p* = 0.16, η^2^ = 0.003 **with** (3): 26.65 (±4.60) dB **without** (30): 30.11 (±1.48) dB	*F* (10, 694) = 0.19, *p* = 0.99; η^2^ = 0.003
Circulatory system	Middle	*F*(10, 1,144) = 20.23, *p* < 0.001; η^2^ = 0.15	*F*(1, 1,144) = 0.35 *p* = 0.55, η^2^ = 0.0003 **with** (22): 16.05 (±2.26) dB **without** (63): 16.78 (±0.81) dB	*F* (10, 1,144) = 0,41, *p* = 0.94; η^2^ = 0.004
	Older	*F*(10, 694) = 12.65, *p* < 0.001; η^2^ = 0.34	*F*(1, 694) = 20.09 *p* < 0.001, η^2^ = 0.05 **with** (19): 33.42 (±3.87) dB **without** (14): 22.39 (±2.86) dB	*F* (10, 694) = 1.32, *p* = 0.22; η^2^ = 0.03
Respiratory system	Middle	*F*(10, 1,144) = 1.76, *p* = 0.06; η^2^ = 0.015	*F*(1, 1,144) = 0.55 *p* = 0.46, η^2^ = 0.0005 **with** (5): 14.64 (±5.48) dB **without** (80): 16.74 (±0.75) dB	*F* (10, 1,144) = 0.39, *p* = 0.95; η^2^ = 0.003
	Older	*F*(10, 694) = 17.19, *p* < 0.001; η^2^ = 0.20	*F*(1, 694) = 3.59 *p* = 0.06, η^2^ = 0.005 **with** (5): 26.60 (±3.53) dB **without** (28): 30.37 (±1.49) dB	*F* (10, 694) = 0.33, *p* = 0.97; η^2^ = 0.005
Digestive system	Middle	*F*(10, 1,144) = 3.81, *p* < 0.001; η^2^ = 0.032	F(1, 1,144) = 3.95 *p* = 0.047, η^2^ = 0.003 **with** (9): 22.23 (±5.55) dB **without** (76): 16.59 (±0.69) dB	*F* (10, 1,144) = 0.14, *p* = 0.99; η^2^ = 0.001
	Older	----	----	----
Muscle-skeletal system	Middle	*F*(10, 1,144) = 25.37, *p* < 0.001; η^2^ = 0.18	*F*(1,1,144) = 66.60 *p* < 0.001, η^2^ = 0.06 **with** (19): 26.12 (±2.40) dB **without** (66): 15.72 (±0.87) dB	*F* (10, 1,144) = 2.29, *p* = 0.01; η^2^ = 0.02
	Older	*F*(10, 694) = 3.26, *p* < 0.001 η^2^ = 0.125	*F*(1, 694) = 1.06 *p* = 0.30, η^2^ = 0.002 **with** (11): 31.32 (±9.83) dB **without** (22): 25.99 (±2.47) dB	*F* (10, 694) = 0.17, *p* = 0.99; η^2^ = 0.005

For details, see text.

The numbers in brackets behind with/without give the included patient numbers. η^2^ effects: < 0.01 no effect, < 0.06 small effect, < 0.16 medium effect, ≥ 0.16 large effect.

## Discussion

With this study, we aimed to investigate if the heterogeneity of tinnitus-related HL can be correlated to single or combinations of non-auditory comorbidities. In summary, we found significant age- and comorbidity-related differences in air conductance pure-tone hearing level measurements of tinnitus patients. Depending on the age group, the number of comorbidities could lead to an increase or even decrease in hearing levels. However, only in older patients, a linear correlation between the number of non-auditory comorbidities and an increase in hearing levels could be found. Moreover, the correlation of maximal HL frequency and tinnitus frequency can only be seen in specific age and comorbidity-number groups. Only some specific non-auditory comorbidity classes showed significant effects on HL (decrease or increase in their hearing ability), adding further variance to the problem of tinnitus heterogeneity.

Generally speaking, the investigation of comorbidities in tinnitus patients is not new, as the heterogeneity of the symptom/disease is a well-known problem (23, 25). Several studies tried to explain the variance in tinnitus patients’ data with auditory or non-auditory diseases (39–41). Some of them focused on psychiatric comorbidities only [e.g. (42–44)], while other combined them with several other factors [e.g. (39, 45)]. In further approaches, the comorbidities were used, e.g., to assess the risk factors of developing bothersome tinnitus (46) or to compare it with other diseases such as chronic pain (47).

Tinnitus itself is most probably induced by the HL (8) but can be modulated by stress or other factors (11) and, in turn, alter the hearing thresholds (13). Nevertheless, not all patients with tinnitus might show a clinically relevant HL of at least 20 dB or have the impairment in the standard clinical testing range of 125 Hz to 8,000 Hz (48). The interaction of the tinnitus—and here especially the TF—with the hearing threshold changes is debated in the field. Some researchers find the TF to be associated with the flanks or inflection point of the HL curve or no association at all [e.g. (49, 50)]; others find it associated with the peak of the HL [e.g. (38, 51)]. Moreover, recent research has demonstrated a significant correlation between tinnitus frequency, loudness, and hearing levels, indicating a tonotopic model underlying the tinnitus percept (52). In this study, we find both findings of the TF and maximum HL frequency to be truly dependent on the age group investigated and the number of non-auditory comorbidities present. In other words, the heterogeneity of the tinnitus studies’ results is a direct outcome of the subpopulation investigated—either knowingly or unknowingly. Moreover, the neuronal basis of tinnitus development and chronification is still under debate (13, 53). Generally, it can be divided into two different stages: the initial and continuing bottom-up changes along the auditory pathway related to the HL and the secondary top-down influences from higher cortical areas and/or the limbic system. Both stages can interact over time with each other and other central mechanisms that can influence perception. As this study deals only with chronic subjective tinnitus patients, the changes in the higher auditory system (54, 55) may be permanent already. Nevertheless, non-auditory comorbidities obviously can affect the neuronal processing. Besides psychological comorbidities [e.g. (56, 57)], systemic diseases such as diabetes mellitus may not only have effects in the inner ear, such as microangiopathy and neuropathy (58), but are also found to have an impact on auditory processing as early as on the level of the inferior colliculus [e.g. (59)] and therefore change pure-tone threshold measurements and the perception of sound significantly. In addition, the treatment of a disease can affect general cognitive and auditory functions, e.g., when antagonizing the glutamate neurotransmitter system (60).

We aimed to select a tinnitus patient collective as homogeneous as possible with respect to the occurrence of auditory comorbidities (cf. Methods) to reduce any HL effects of these diseases. We then separated the patients into three different age groups. Nevertheless, we cannot rule out that some patients might have undetected or unreported auditory or non-auditory comorbidities, as we performed the analyses on the data the patient files provided. The analyses of the tinnitus-related parameters, such as frequency and loudness, did not show any difference between the patients of the three age groups. These parameters were also not affected by the number of non-auditory comorbidities, while the number of comorbidities did trivially depend on the patients’ age. Nevertheless, we did find a significant TF distance dependency of the peak of the HL relative to age and number of non-auditory comorbidities (cf. above). This could explain the differences found in different tinnitus patient groups in other studies, where the TF did or did not match the maximum HL (34, 61–63). Furthermore, the trivially age-dependent mean HL across all frequencies also shows further dependencies on the number of non-auditory comorbidities. In other words, the effects cannot be simply described as related to presbycusis only. In a vicious circle, the increased number of such comorbidities might increase the individual stress, which in turn might increase the vulnerability for further comorbidities and vice versa. This combinatory effect of age, possible stress, vulnerability, and number of comorbidities might explain differences in reported effects on HL in tinnitus patients as well [e.g. (64, 65)] and, therefore, might add to the explanation of at least a part of the overall heterogeneity in the data reported in the literature.

The attempt of the investigation of the single non-auditory comorbidity category effects on the HL has to be seen as a first analysis step. Especially, as in some cases, the number of patients with or without a given comorbidity was limited; this fact can also be seen in the partially different outcome of p- and η^2^- values in the single tests. A more fine-grained investigation of single diseases in a larger patient collective and a comparison with non-tinnitus patients with the same non-auditory diseases might give much more insight in possible mechanisms on the effects of HL severity or the development of tinnitus itself. Nevertheless, our approach to explaining a part of the variance of audiometric data in tinnitus patients revealed some candidate non-auditory comorbidity categories where such investigations might be fruitful. In our analyses, especially the diseases of the endocrine or metabolic system had a strong—to our surprise positive—effect on the severity of the HL. This could indicate that there is a causal relationship here rather than a simple aging effect. One possible explanation of this effect could be the patients receiving medication (e.g., hormone substitutes), which in turn reduce stress (66) and therefore changes HL and/or the tinnitus percept. Alternatively, e.g., in hyperlipidemia patients, the treatment with statins may also lead to a positive change in HL and tinnitus percept (67). In line with reports about key cardiovascular risk markers being strongly associated with tinnitus and may aggravate cochlear ischemia or central auditory dysfunction (68), negative effects on hearing ability could be observed in patients with circulatory diseases in the elderly. This negative effect was also observed in such patients with muscle-skeletal system diseases as well as in patients with digestive system diseases in the middle-aged tinnitus patients. As the patients’ disease categories of the non-auditory comorbidities cover a wide range of individual diseases with different kinds of expression strengths within each disease, we refrain from providing any mechanistic or pathological explanation—but compare (28, 31), e.g., where correlations of HL extend and specific comorbidities such as hypertension and diabetes mellitus are discussed—although this is rather a phenomenological description. On the other hand, especially the comorbidities regarding mental health, which are seen by many researchers and physicians as most relevant for tinnitus intrusiveness [e.g. (69)], do not show any significant effects on hearing thresholds. This is in line with reports that hearing-impaired patients with similar pure-tone audiometries can have significantly different tinnitus distress (70, 71) even when their other tinnitus parameters, such as TF and TL, are quite similar.

The limitations of this study are the relatively low number of patients in some comorbidity classes—especially in the young patients group—and the lack of a comparable number of non-tinnitus patients with the respective comorbidities. These data were extracted from patients’ files, which included medical and self-reported comorbidities; possible treatments of the individual diseases were not taken into account. Additionally, we did not use a mean hearing threshold change for the patients but the HL of the tinnitus-affected ears. This resulted in the fact that for the case of binaural tinnitus, the comorbidity classes of the patients were used for both ears in the independent ANOVAs, possibly leading to an increased weight of the single binaural tinnitus patient in that analysis. Nevertheless, any given comorbidity class could affect both ears and therefore this procedure might still be correct. Furthermore, we cannot rule out that not every comorbidity was included in the patient files for this retrospective study. In some cases, we might have underestimated the number of comorbidities. As we nevertheless saw a change of HL from “no comorbidity” to at least one of the “with comorbidity” classes, we think that our main conclusion—different comorbidities have different influences on hearing thresholds in tinnitus patients—is still valid.

## Conclusion

In conclusion, our results show the need for a more comprehensive, multidisciplinary approach to tinnitus management in the clinics, taking into account co-existing comorbidities. For research on the neuronal basis of tinnitus development and chronification in humans, the same is true. One should be aware of the different effects non-auditory comorbidities might have on the results and therefore carefully select and evaluate the patient and control groups to help to avoid wrong conclusions. For understanding the effects of the different comorbidity pathologies on the hearing thresholds of tinnitus patients, much more focused prospective studies have to be performed. One candidate non-auditory comorbidity category includes diseases of the endocrine or metabolic system, which provides surprising results and should be investigated more thoroughly in prospective clinical studies.

## Data Availability

The raw data supporting the conclusions of this article will be made available by the authors, without undue reservation.
